# Selection criteria for use and conservation priorities for plant species in a Quilombola community in Baixada Maranhense, Brazil

**DOI:** 10.1186/s13002-025-00798-w

**Published:** 2025-09-26

**Authors:** Thauana Oliveira Rabelo, André Luiz Borba do Nascimento, Eduardo Bezerra de Almeida

**Affiliations:** Programa de Pós-Graduação em Biodiversidade e Conservação (PPGBC), Universidade Federal do Maranhão (UFMA), São Luís, Maranhão Brazil

**Keywords:** Amazon, Ethnobotany, Perception of abundance, Conservation unit

## Abstract

**Background:**

The Amazonian biome is home to a vast diversity of plant species that are of fundamental importance to traditional communities such as the Quilombolas. These communities use different criteria to select and prioritize species for use. This study focused on the perceptions of these communities regarding the availability of medicinal, food, and timber plant species, with the aim of identifying priority resources for conservation and assessing the risks associated with the collection of these resources in the Queluz Quilombo, Anajatuba, Maranhão State. To this end, the following hypotheses were tested: Useful plants perceived by informants as more abundant locally will be more multifunctional; (II) more versatile; (III) more frequently recalled by informants; and (IV) there is a difference in collection sites depending on the use category.

**Methods:**

Between March 2022 and December 2023, 75 out of 105 families residing in the visited communities agreed to participate in the study and were interviewed using semi-structured questionnaires that included socioeconomic questions and free lists. Plants were assessed based on their perceived abundance in the area. Generalized Linear Models were used to examine the relationship between perceived abundance, species versatility, and their salience by category. In addition, percentages were calculated for collection sites and the structure of the most affected plants within each category.

**Results:**

Multifunctionality and versatility food did not significantly affect the perceived abundance of useful plants; however, versatility in medicinal and timber use was found to be significant (*p*-value: 0.012* e *p*-value: 0.011*, respectively). Proximity of resources to homes was a key indicator of abundance for food plants, while effectiveness and quality were the primary factors for medicinal and timber species. Timber species such as Paparaúba (*Simarouba* spp*.*), Sabiá (*Mimosa caesalpiniaefolia* Benth.), Pau D’arco (*Handroanthus albus* (Cham.) Matto), Cedar (*Cedrela* spp.), and Jenipapo (*Genipa americana* L.) were identified as being under greater use pressure. However, local factors have mitigated the risks associated with collecting these resources. The study found that medicinal and food plants are mainly collected in backyards (87.07%), while timber plants are more commonly gathered from the forest (74.14%). The parts of plants most affected differ by category, with leaves (97.14%) and fruits (77.73%) being primarily collected from medicinal and food plants, respectively, and stems (100%) from timber plants.

**Conclusions:**

These findings provide valuable insights for the development of conservation strategies and the sustainable management of plant resources within local communities. The results emphasize the need to consider both biodiversity and the socioeconomic and cultural dimensions involved in the use of these natural resources.

## Background

The selection of useful species within traditional communities is not a random process. Moerman [1] argues that humans, like other animals, carefully select the resources they integrate into their socioecological systems, and shows that foraging follows specific patterns. For human groups, plant use extends beyond mere subsistence to include criteria such as cultural value, adaptive memory, and emerging intercultural influences that contribute to the incorporation of new knowledge and plant species [2].

To investigate the criteria that communities use to define useful species, as well as the changes that occur in these choices and in patterns of resource use and collection, numerous ethnobotanical studies have focused on assessing and relating individuals’ knowledge and perceptions of the flora [2–8]. These factors include the appearance, temporal, economic, and geographic availability of resources [2, 9], socioeconomic factors [10, 11], and disturbances in socioecological systems [12, 13].

In areas that are anthropized or undergoing fragmentation, the understanding of the flora is influenced by the memories of residents who occupied the region prior to urbanization [10]. Urban expansion often reduces or eliminates traditional communities of access to local flora through habitat destruction, biodiversity loss, and the privatization of collection areas, leading to a dependence on industrialized products and the erosion of ancestral knowledge among younger generations [14–16]. In the literature, this process is discussed as one of the main factors altering the dynamics of species knowledge and use [17].

Recognizing the analysis of community knowledge as a tool for identifying priority species and areas for conservation, ethnobotanical research has used various approaches. One of them is the analysis of collection methods, which considers the plant structures affected during the collection process, and assesses the risks associated with the survival and reproduction of plant species, as well as information such as species abundance and local importance [18–20].

It is also essential to analyze the specific criteria used for selecting plant species according to their use categories. For timber, for instance, the selection depends on the intended use: For firewood, local availability is the main factor, while for construction purposes, higher quality and durability are required, leading to the collection of wood in more distant areas such as forested zones [6].

In contrast, more specialized uses, such as medicinal and food plants, follow different selection criteria, and harvesting is mostly done around people’s homes or nearby areas. Medicinal plants are chosen based on traditional knowledge passed down through generations, considering attributes such as taste, smell, texture, and chemical effectiveness. In the case of food plants, selection is influenced by taste, aroma, appearance, physiological effects, cultural values, and the absence of social or economic prejudice [20]. Moreover, there is a preference for species that also have medicinal value, highlighting the importance of multifunctionality [21].

Some studies have also shown that species with multiple uses are under greater pressure in the environment [22], suggesting that variables such as versatility and multifunctionality could be used to assess whether use pressure is affecting the plant composition of an ecosystem [23]. These variables and species recall may be sensitive to the perceived abundance of resources in the environment, thereby influencing how they are used [24].

The choice to use a particular plant may be intrinsically related to its ecological importance [9]. In other words, plants that are more visually prominent due to their density in the landscape are more likely to be experimented with and incorporated into local cultural practices. Maximization theory supports this perspective, positing that the frequency of resource use is closely related to its accessibility and visibility in the environment [25]. Therefore, when analyzing the perception and selection of plant species, it is essential to consider the collection site. The distance between the collection site and inhabited areas may influence access to and preference for certain plants [26], thus enriching our understanding of plant use patterns in specific cultural and environmental contexts.

Among the studies conducted in Maranhão, floristic research has briefly addressed the interactions between Quilombola knowledge and flora conservation [27, 28]. These studies highlight the composition of the flora in quilombos and provide insights into the relationship between the decline of vegetation, the fragmentation of territories, and the reduction of the repertoire of plants cited by these communities, but without in-depth analysis.

Studies that analyze the criteria for the use of flora by traditional communities are needed, especially in areas such as the Baixada Maranhense. These regions are environmental protection zones that are home to numerous Quilombola communities, whose way of life and relationship with the flora have been significantly affected by anthropogenic changes [29, 30].

Quilombola communities in the Baixada Maranhense have an intimate and multifaceted relationship with the plant species surrounding their territories. Uses for food, medicine, construction, and handicraft are generally the main reasons for the exploitation of timber and non-timber resources in these communities, and they can be key indicators of impacts and use pressures on local communities [31].

The aim of this study was to analyze the perceptions of the Queluz Quilombo community regarding the availability of medicinal, food, and timber plant species, with the aim of identifying priority resources for conservation and assessing the risks associated with the collection of these resources. In addition, it examined the collection sites and methods used to harvest these resources. Unlike the traditional concept of versatility [18], which focuses on a single category of use, this study addresses three distinct categories. The ecological variable of multifunctionality considers the frequency with which a plant is mentioned in these categories. Meanwhile, the variable of versatility was used to analyze the different uses of each plant within each specific category.

Analyzing the perceived abundance of plant resources and their forms of use is crucial for understanding local use pressures on these resources locally and for formulating better conservation strategies. Some ethnobotanical studies have evaluated similar parameters to identify priority species for conservation [19, 22–24], including the analysis of plant parts used and collection methods to assess the risks associated with resource exploitation. Therefore, this study explores the relationship between plant use and ecological and cultural factors, contributing to a broader understanding of exploitation pressure that can guide targeted and effective conservation strategies.

In light of the above, we propose the following hypotheses: (I) Useful plants perceived by informants as more abundant locally will be more multifunctional; (II) useful plants perceived as more abundant will be more versatile; (III) useful plants perceived as more abundant will also be more frequently recalled; and (IV) there is a difference in collection sites depending on the use category. We expect that our hypotheses will be confirmed regardless of the use categories.

## Materials and methods

### Study area and socioeconomic characteristics

**Fig. 1:**
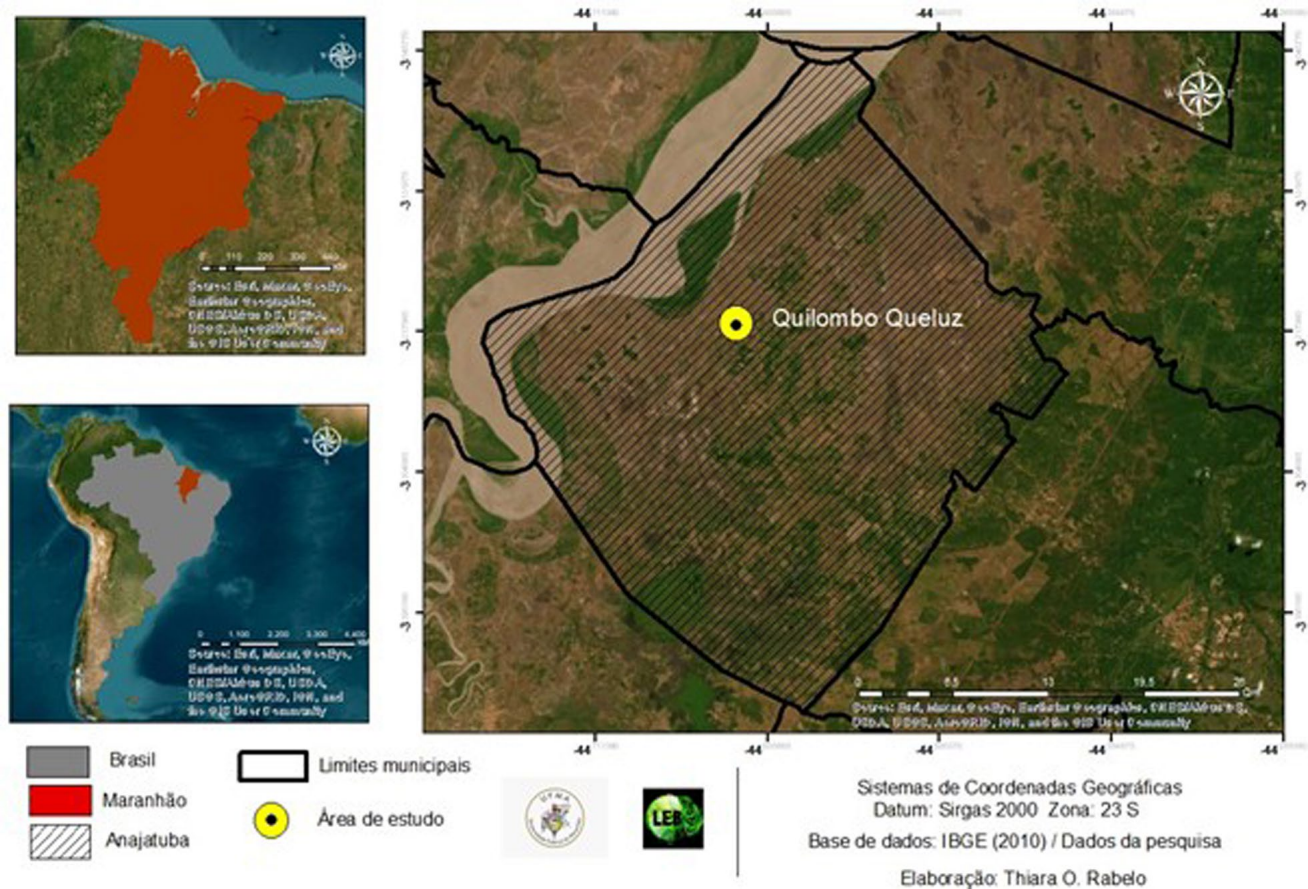
Location Map of the Municipality of Anajatuba, Maranhão, Northeast of Brazil

The study was conducted in the municipality of Anajatuba, located in the micro-region of Baixada Maranhense in the state of Maranhão, Northeast Brazil (Fig. 1). The municipality covers an area of 1117 km^2^ and has a population of 25,294 inhabitants, with a population density of 22.64 inhabitants/km^2^ [32].

Within this community, approximately 16,704 inhabitants identify as Black, representing 72.26% of the population. Most of these people are organized in 27 Quilombola communities recognized by the Palmares Cultural Foundation [33]. This is due to the historical recognition of the municipality’s predominantly Black heritage. Beginning in the 17th century, the state’s agricultural export economy attracted a large influx of enslaved Africans, who mixed with the indigenous population to form a distinct culture with diverse characteristics [34].

The municipality’s hydrographic network includes the Mearim River and its tributaries. Due to its low latitude, the climate is tropical, hot, and humid, with an average temperature of 27 °C, low seasonal variability, a temperature range of about 2 °C, and annual rainfall ranging from 1800 to 2000 mm [35].

The vegetation in this area is typical of an ecotonal region, with characteristic species such as the Brazil nut tree (*Bertholletia excelsa* Bonpl., Lecythidaceae), the gameleira (*Clusia burchelli* Engl., Clusiaceae), the embaúba (*Cecropia* spp., Urticaceae), the cedar (*Cedrela fissilis* Vell., Meliaceae), and the babaçu (*Attalea speciosa* Mart. ex Spreng, Arecaceae) [36].

The municipality’s structure consists of rural settlements and a municipal center located on the banks of the flooded fields, where human expansion and settlement efforts are concentrated [35]. Although this area is considered urban, it does not have the typical spatial configuration of a city with defined neighborhoods. However, it does provide essential infrastructure and services for the rural population, including hospitals, shops, schools, and banks.

The focus of this study is the Queluz Quilombo, located 38 km from the center of the municipality. This area was certified by the Palmares Foundation, as established by Ordinance No. 221 of December 20, 2013. According to the latest census, 105 families belong to the Quilombola association.

The community preserves a rich Afro-Brazilian cultural heritage, expressed in popular Catholic religious manifestations such as the Feast of the Holy Spirit and the *Tambor de Crioula*. The latter is practiced in devotion to saints such as Saint Benedict and Our Lady of the Conception, involving dance, singing, percussion with handcrafted drums, and the practice of the “punga,” which consists of a symbolic bumping of navels and serves as a form of greeting and invitation [37]. These cultural practices are intertwined with the local way of life, marked by traditional woodcraft work, including the making of mortars, troughs, furniture, and fishing traps, in addition to the production and commercialization of manioc flour, a collective activity that is central to the livelihood of 91 families in the region. The cultivation and processing of manioc, carried out in traditional flour houses (*casas de farinha*), preserves ancestral techniques, serving both as a dietary staple for the community and as a product for sale [38].

Although it is one of the communities farthest from the urban center of Anajatuba, its internal organization and visibility—enhanced by the presence of mining train tracks that cross its territory—provide it with greater support. According to the IBGE [32], the municipality’s social and economic indicators reveal profound inequalities in support and resources between urban and rural areas. The rural area, where the Queluz Quilombo is located, has a high illiteracy rate and limited access to sanitation services. However, there is a nearby health center open until 4 p.m. and a municipal primary school.

This community preserves one of the largest forest areas in the municipality of Anajatuba, characterized by a rich transitional vegetation between the Cerrado and the Amazon biome, including babaçu palm forests, gallery forests, and seasonally flooded grasslands [36]. According to the IBGE [37], a significant part of the population depends on agricultural activities, which play a crucial role in supporting the center of the municipality.

### Data collection

The ethnobotanical studies were carried out between March 2022 and December 2023 with household heads (men and women over 18 years of age) in the Queluz Quilombo who agreed to participate in the study. Of the 105 families residing in the visited communities, 75 agreed to participate and were included in the sample, with one person interviewed per household, preferably the individual recognized as responsible for the home. Data were collected using semi-structured interviews, divided into two sections: the socioeconomic questionnaire and the free list. The socioeconomic questionnaire included questions about gender, age, length of residence, education level, and occupation to characterize the profile of the community.

In the second phase, the free list technique [39] was used to identify the plants used in the communities for food, medicinal, and timber purposes. After completing the list provided by the informants, the new reading method [39] was applied. This method added information such as the place of collection, the part used, and the form of use for each plant in order to extract as much information as possible. The species mentioned were rated on a scale of 1–5, based on the interviewees’ perception of their abundance in the area, with 1 representing “not found much” and 5 representing “found a lot.” Before beginning the free list, respondents were reminded to list only the species they actually use.

This study was approved by the Human Research Ethics Committee (CEP) of the University Hospital of the Federal University of Maranhão (HU-UFMA), registered in the Brazilian Platform (Opinion no. 4,679,396). Authorization to conduct scientific activities in the Baixada Maranhense EPA was also obtained from the Environment Department of Maranhão (process no. 2003260020).

### Data analysis

The interview data were organized in Excel spreadsheets to create a database for this research. The data were filtered to ensure quality, considering only those species mentioned by the interviewees for which there was information on their use or the perceived abundance was available. Species without this information were discarded.

Multifunctionality data were calculated for the remaining species by summing the use categories in which each plant was mentioned. Average perceived abundance values by the informants were also calculated by summing the scores given to each plant by each respondent in each category and dividing by the total number of respondents who provided scores. Species were also assessed for their versatility, with this parameter calculated for timber and food species by summing the total number of unique uses attributed to each species. For medicinal species, the sum of the total number of unique diseases treated with the use of the species was considered [17].

A Generalized Linear Model (GLM), Poisson family, was used to assess the relationship between perceived abundance (predictor variable) and the versatility of species in the food, medicinal, and timber categories (response variable). The analyses were performed in R software, version 3.4.3 (R Core Team 2017), using the “scales,” “glmnet,” and “lassopv” packages.

To identify the most important plants in the community, the salience of these species was calculated for the three categories of use using R software, version 3.4.3 (R Core Team, 2017). The salience calculation followed Smith’s Salience Index, with analysis took into account the order and frequency with which each informant mentioned the species, as recorded using the free list, as described by Kutal [40]. The influence of perceived abundance on salience across the use categories was tested using Generalized Linear Models (GLMs) with quasibinomial distribution, due to the continuous and proportional nature of the data.

In all models, the predictor variable was the abundance perceived by the participants. The response variables were: (I) multifunctionality (number of use categories attributed to the plant), (II) medicinal versatility (number of distinct uses within the medicinal category), (III) food versatility (number of distinct uses within the food category), and (IV) timber versatility (number of distinct uses within the timber category).

Percentage analyses were performed to determine the distribution of collection sites (house, forest, and farms) across the different categories of use. In addition, the frequency of use of different plant parts (bulb, bark, fruit, leaf, stem, latex, aerial parts, whole plant, root, sap, and seed) within each use category was evaluated. This method allows for the identification of preferred collection sites and the most commonly used plant parts in each category, following a similar approach to the previous studies in the area [41].

## Results

### Multifunctionality and Versatility as predictors of the species’ perceived abundance

The linear regression models revealed distinct relationships between plant abundance and the evaluated variables (Table 1). According to the analysis, no significant relationship was found between multifunctionality and perceived local abundance (*p*-value: 0.801). This suggests that multifunctional plants are not necessarily perceived as more abundant in this model.

**Table 1:** Analysis of the Generalized Linear Model relating variables such as versatility and multifunctionality to the perceived abundance of plant species recognized by the Quilombola community of Queluz-Anajatuba, Maranhão, Northeast Brazil

Models	Estimated	Standard error	T value	*P* value	AIC value
*Modelo 1—Multifunctionality*					530.72
Intercept	0.1521	0.1357	1.12	0.263	
Perceived abundance	0.0027	0.0408	0.067	0.947	
*Modelo 2—Medical versatility*					967.69
Intercept	− 0.5387	0.1588	− 3.392	<0.001	
Perceived abundance	0.2344	0.0428	5.481	<0.001*	
*Modelo 3—Food versatility*					773.28
Intercept	0.0317	0.1428	0.222	0.824	
Perceived abundance	0.0153	0.0426	0.358	0.72	
Modelo 4—Timber versatility					862.45
Intercept	− 1.2566	0.2075	− 6.056	<0.001	
Perceived abundance	0.3348	0.0538	6.226	<0.001*	

Significance Level: *p* > 0.05; *

Similarly, Model Food Versatility (Table 1) showed no significant association between food versatility and perception of local abundance (*p*-value: 0.708). Thus, this model does not support the idea that plants with food-related uses are perceived as more abundant. However, in Models Medical versatility and Timber Versatility, a significant positive association was found between medicinal and timber versatility and the perception of local abundance (*p*-value: 0.012* and p-value: 0.011*, respectively). This suggests that medicinal and timber plants with greater versatility are those perceived to be among the more abundant.

These results contradict the expectation of a positive relationship across all three use categories, as no significant effect was observed for food plants. This suggests that perceived abundance may vary by use category, possibly due to factors specific to each category. Therefore, a separate analysis of the use categories may be necessary to better understand how these factors influence the perceived abundance of plants.

Overall, the results suggest that perceived abundance is sensitive to medicinal and timber versatility, and this correlation may be an explanatory factor within these use categories. Conversely, multifunctionality and food versatility do not show significant associations in these models. The lack of association in the food category underscores the complexity of this dynamic and highlights the importance of analyzing use categories separately.

### Species most remembered by the community and their relationship with use pressure

Linear regression models were also fitted to assess the relationship between cultural salience and different use categories (medicinal, food, and timber) (Table 2). Regarding “Food Salience,” the estimated coefficient is statistically significant (*p*-value = 0.001). Similarly, for “Medicinal Salience” variable, the estimated coefficient is statistically significant (*p*-value = 0.002). This suggests that the food and medicinal plants that are most remembered by the informants tend to be perceived as more abundant.

**Table 2:** Analysis of the Generalized Linear Model relating variables such as salience to perceived abundance of plant species recognized by the Quilombola community of Queluz-Anajatuba, Maranhão, Northeast Brazil

Models	Estimated	Standard error	T value	*P* value	AIC value
*Modelo 1—Medicinal Salience (QuasiBinomial)*					15.71
Intercept	− 4.428	0.2831	− 15.644	<0.001	
Percepção de Abundância	0.3063	0.0999	3.065	0.002*	
*Modelo 2—Food Salience (QuasiBinomial)*					20.67
Intercept	− 4.3878	0.3527	− 12.44	<0.001	
Percepção de Abundância	0.5259	0.1397	3.764	<0.001*	
*Modelo 3—Timber Salience (QuasiBinomial)*					19.44
Intercept	− 3.1317	0.4951	− 6.325	<0.001	
Percepção de Abundância	0.0233	0.1205	0.193	0.847	

Significance Level: *p* > 0.05; *

This rejects the initial hypothesis is refuted, as the expectation was for a positive relationship between salience and perceived abundance was expected across all use categories. These results suggest that while local uses may be sensitive to perceived abundance, this influence may vary according to factors specific to each use category.

For the “Timber Salience,” the estimated coefficient is not significant (p-value = 0,847), indicating no significant relationship between this variable and the perceived species abundance.

### Variation in resource collection sites by use category

The data obtained provide interesting insights for the analysis of Hypothesis IV. Table 3, which shows the locations where resources are collected, reveals significant variation in collection locations depending on the use category of the plant. For example, medicinal plants are mainly collected in backyards (90.38%) and forests (7.05%). In contrast, food plants are mainly collected in backyards (66.37%) and on farms (25.05%). Timber plants, on the other hand, are mainly collected in forests (74.14%).

**Table 3:** Resources collection sites by the Quilombola Community of Queluz-Anajatuba, Maranhão, Northeast Brazil

Collection sites	Medicinal plants (%)	Food plants (%)	Timber plants (%)
Backyards	90.38	66.37	23.28
Farms	1.92	25.05	1.72
Fairs	0.64	2.96	0.86
Forests	7.05	5.62	74.14

The data in Table 3 suggest a relationship between the plant use category and the collection locations. There seems to be a preference for collecting medicinal and food resources in more accessible areas, such as backyards. Meanwhile, timber plants are mainly collected in natural environments such as forests. This finding supports the hypothesis that the locations where resources are collected differ depending on the use category of the plant.

Looking at Table 4, which details the plant structures used in the three use categories (medicinal, food, and timber plants), there is a clear preference for certain plant parts in each category. For example, leaves are predominantly used for medicinal purposes, representing a significant proportion of 80.75%. Conversely, fruits are the most commonly used part of food plants, accounting for 74.83%.

**Table 4:** The plant structures used in the three categories of use, by the Quilombola community of Queluz-Anajatuba, Maranhão, Northeast Brazil

Plant parts	Medicinal plants (%)	Food plants	Timber plants
Bulb	0.21	0.29%	0
Bark	3.31	0	0
Fruits	3.31	74.93%	0
Leaves	80.75	13.59%	0
Stem	0.62	1.44%	100%
Latex	0.83	0.10%	0
Aerial parts	3.31	0.48%	0
Whole plant	1.24	0.10%	0
Root	4.14	6.51%	0
Sap	0.21	0	0
Seed	2.07	2.58%	0

In contrast, the stem is the only part collected from timber plants, representing 100% of the use. The concentration of timber plant collection in forests and the exclusive use of stems, which directly affects the individuals collected, may indicate a concern for the conservation of these resources.

## Discussion

### Multifunctionality as a predictor of species’ perceived abundance

The study found that multifunctionality did not significantly influence the perceived abundance of plant species, which is contrary to expectations based on the hypotheses tested regarding species selection for use [42, 43]. This finding suggests the need to consider other factors not analyzed in this study that may help explain the results. One such factor could be the overlap of use categories, which is generally associated with greater efficiency in resource use and implies lower energy expenditure [44]. However, recent studies [45, 46] suggest that people may prefer species with better chemical efficiency or those that produce higher quality wood. In these cases, the reduced energy expenditure associated with multifunctionality may not be sufficient to compensate for lower efficiency, leading to a lower valuation and perceived abundance of multifunctional species.

Another possibility is that plants that are recognized for their multifunctionality may exhibit pronounced versatility in a particular use category, making them more conspicuous and thus perceived as more abundant. This dynamic suggests that plants with multiple uses are more easily perceived in the environment [47].

When analyzing the use categories in this study, food and medicinal species were found to have more individual uses attributed to them. This finding suggests that individuals may have already maximized the use potential within a given category [48]. Cruz et al. [11] observed a similar pattern, where a food plant recognized for its palatability and ease of cultivation was used in a greater number of preparations. Similarly, medicinal plants that are perceived to be effective, especially those that treat multiple health problems, are used extensively for medicinal purposes [49]. In this sense, multifunctionality may limit the general perception of a plant’s abundance, as the average abundance reflects contributions from each category. This suggests that although multifunctionality is present, it may not be the dominant factor influencing the perception of local abundance. Instead, the versatility and richness of plant species in the Maranhão Amazon may play a more important role.

This finding differs from most studies on multifunctionality conducted in Brazil, which focus on semi-arid areas [50, 51]. According to Albuquerque et al. [44], arid environments have lower species density and abundance, and climatic variations limit the temporal availability of resources. These characteristics influence the more unstable environments, promoting generalized use and species multifunctionality.

It is important to consider the specific context of the study site, located in an area influenced by the Amazon, with conservation measures in place, two well-defined seasons, and less climatic and temporal variability [52]. In these areas, there is a pronounced richness and diversity of useful plants with greater temporal availability, leading to more stable environments and specialization in plant use [48, 53]. This may have diluted the perception of abundance for multifunctional species.

### Versatility as a predictor of the species’ perceived abundance

The lack of association between perceived abundance and food versatility suggests that variation in a plant’s food utility may not be a determining factor for informants in their perceptions of abundance. This finding supports Leonti et al. [54], who argued that the criteria for species selection vary within each cultural system, depending on specific characteristics and influences.

The previous studies, such as those by Rozin [55] and Nascimento et al. [56], have shown that species preference for food can be influenced by factors such as taste, appearance, physiological effects, cultural influences, and nutrient content. The distance from which resources are collected also plays a role in species preference, as reported by Ladio and Lozada [57]. They found that resources that are abundant closer to home tend to be used more frequently used by communities.

In addition, Ladio and Rapoport [58] observed that species preferred for food are often managed closer to home in order to optimize the energy expended in searching for resources in the forest, making them more available in times of scarcity. This finding is consistent with our data, where the food plants with the highest perceived abundance—mango, tanja lemon, and joão gome—are predominantly grown in people’s backyards. The species with the greatest dietary versatility—cassava, babaçu, and corn—are grown on farms near or in the forest. These plants often form the basis of the diet in traditional communities in Maranhão. In addition to being conventional, these foods play a fundamental role in the diet and subsistence of these communities [29, 31]. Therefore, the availability of nearby resources and the communities’ dependence on these food resources significantly influence the preference and perceived abundance of food species.

However, the versatility variable does explain the perceived abundance within the medicinal and timber categories. More abundant species are likely to be tested for more uses. According to Caetano et al. [59], the availability and effectiveness of medicinal plants play a crucial role in determining the preferred and most versatile species, making these factors key explanatory variables for species selection in the medicinal category.

In this study, the plants with the greatest medicinal versatility were mastruz (*Dysphania ambrosioides* (L.) Mosyakin & Clemants), pitoco (*Pluchea sagittalis* (Lam.) Cabreras), thick leaf mint (*Plectranthus amboinicu*s (Lour.) Spreng’s), peppermint (*Mentha piperita* L.), lemon balm (*Melissa officinalis* L.), and boldo (*Gymnanthemum amygdalinum* (Delile) Sch.Bip. ex Walp.). These species, which are used to treat a variety of ailments, are typically grown in flowerbeds and backyards. Mastruz is the species with the greatest medicinal versatility in the Queluz community. This plant is classified as exotic and thrives in temperate and tropical climates [60]. It has also been reported as the most representative species in several studies on the medicinal potential of Quilombola communities. Mastruz is used to treat various ailments, including flu, bone fractures, inflammation, and parasitic diseases [61, 62]. Notably, other species such as pitoco, thick leaf mint, peppermint, lemon balm, and boldo, which are also notable for their versatility and abundance, are primarily used to treat respiratory ailments. This may be related to the post-pandemic context, where the demand for medicinal plants that support respiratory health may have increased, influencing the perception of their versatility in the region [40]. The Maximization theory, which posits that maximizing the benefits of a resource leads to maximizing its uses, explains these choices [25].

The results for timber versatility highlight species such as paparaúba, pau d’arco, sabiá, jenipapo, and cedar, which were also identified as woods with good durability. These choices can be explained by the local availability of these species and the quality of their wood. Studies show that the selection of wood species depends on their availability and quality in the region [53].

However, the lack of ecological information made it impossible to confirm the local abundance of these species. Nevertheless, the perceived abundance of timber species suggests that the plants considered most abundant in the ecosystem and recognized for their quality are used by the community for a wider range of purposes [63].

### Plants most remembered by the community

A total of 238 ethnospecies were identified in the free list and included in the Salience Analysis (ISC). The most culturally important species for food use were tanja lemon (0.0066), joão gome (0.0004), mango (0.0004), mucajuba (0.0003), and yam (0.0001). In the case of medicinal species, the ones that stood out were Vassourinha (0.0009), Castor bean (0.0024), Purple vinegar (0.0095), and Nettle (0.0095).

These plants occupy a prominent place in the community’s collective memories, which reveals their cultural and utilitarian importance in daily life, as reinforced by Pedrollo et al. [64]. These species stand out not only because they were mentioned frequently, but also because they were among the first to be remembered, reinforcing their cultural importance within the community [65]. Furthermore, these plants had a high perceived abundance, which may be due to their general utility in meeting the primary needs of the informants [66]. People may prefer species that are readily available or abundant to satisfy their immediate needs, such as hunger and treating diseases [67].

This observation supports the hypothesis that most of these plants are located near homes, consisting mainly of ruderal species used for medicinal purposes and managed species grown in backyards, which may be for food and medicinal use [57], further suggesting that perceived abundance is related to the food salience and medicinal salience of species in this category. However, this hypothesis was not supported for timber plants. This category often requires more specialized species, where environmental availability is a critical factor for their effective use. Therefore, the species considered most important are likely to be the higher quality for a given technological purpose [68]. Local informants emphasized this specialized use by identifying different timber species for specific purposes, such as roofing, house support, and furniture.

This perspective can also be explained by adaptive memory theory, which suggests that we tend to remember information that is essential for survival or that we have used recently [69]. As such, the most prominent timber species would be those that have been formalized in recent years or have demonstrated superior performance in terms of durability, weight, and heat resistance [46].

### Variation in resource collection sites by use category

Resource collection sites provide valuable insights into the management and conservation of the local flora. In this study, crops were distributed across natural areas, such as forests, and production areas, such as farms and backyards. Although the literature indicates that forests are the main source of medicinal plants [70, 71], this study found that most of medicinal plants are primarily collected from backyards, with some opportunistic collections in forests. This preference may be related to the ease of access and the transmission of medicinal plant cultivation practices within domestic environments, which can contribute to the conservation of these species.

Studies conducted by traditional communities in Amazonian regions reveal that backyards contain a high diversity of medicinal species, which significantly contributes to plant domestication and management while playing a crucial role in food sovereignty [72–74]. In contrast, food plants in this study are predominantly collected from backyards, followed by farms, and to a lesser extent, from fairs and forests. This indicates that food plants are often cultivated in areas associated with traditional agricultural practices of the state’s Quilombola communities [74].

Another method of acquiring food resources mentioned by informants is through fairs and supermarkets, which may reduce the pressure on native species. This practice aligns with findings from studies conducted in the Piagaçu-Purus Reserve in the Central Amazon, where a shift toward industrialized foods among younger generations, coupled with the fatigue of maintaining farms, has led to increased reliance on purchased food items [75].

Timber species are predominantly collected from forests (74.14%), suggesting a greater potential risk to their conservation. Intensive exploitation of these areas could result in habitat degradation and a decline in timber plant populations [76]. However, the collection of some species, such as mango and murici trees, from backyards may indicate a sustainable management practice within inhabited areas, mitigating the impact on local forests.

The collection patterns also varied according to the parts of the plants most frequently used in each category. For medicinal plants, leaves were the most commonly collected part, representing 80.75% of the occurrences. In contrast, fruits were the predominant part collected from food plants, accounting for 74.83% of the occurrences. In the studied community, the collection of medicinal and food plants poses a lower risk to conservation, as it typically involves aerial and transitory structures. This contrasts with studies in semi-arid regions, where medicinal plant collection often targets perennial structures such as bark and roots, posing additional conservation challenges [24, 77].

According to the interviews, the stem is the only part used from timber plants, accounting for 100% of the occurrences, which increases the conservation risk. Timber collection in forests, where exploitation can be intense, is detrimental to the species’ conservation, as it often involves complete removal of the stem, leading to the death of the individual [74, 78].

As a result, timber species such as paparaúba (ISC: 0.4155), sabiá (ISC: 0.3153), pau-d’arco (ISC: 0.1632), cedar (ISC: 0.078), and jenipapo (ISC: 0.0734) are highly versatile and culturally salient in the community, indicating significant use pressure. However, these commonly chosen species for collection are also the most abundant in the survey. While intensive collection may not pose an immediate threat, reducing their abundance or vegetative cover could negatively impact local biodiversity.

Paparaúba plays an important role in the forest canopy structure, reaching large sizes and being preferred for traditional civil construction and furniture making. Sabiá is a fast-growing pioneer legume, commonly found in open and regenerating areas, and is used for fence posts, furniture, and internal house structures. Pau-d’arco is a species that blooms during drier periods and is cultivated in the region as an alternative for furniture production, roofing structures, and fencing. Cedar has moderate growth and is known for its high-quality timber, which is preferred for sale and valued in the production of furniture, religious artifacts, kitchen utensils, and civil construction. Jenipapo, in turn, is a sub-canopy fruit tree with ecological importance for attracting wildlife; its fruit is used for food, and its wood is used in construction and furniture making [23, 53].

It is worth noting that many houses in the community are built with masonry materials, which may help reduce pressure on timber resources. Future studies should provide a more in-depth ecological analysis of species richness and density in the area. Additionally, it is essential to correlate local community perceptions with ecological parameters to develop a more comprehensive and integrated understanding of the relationship between environmental aspects and traditional biodiversity use practices.

## Conclusions

Based on the analyzed data, multifunctionality does not appear to be a significant criterion for meeting the needs of the communities in the studied region. This is likely due to the great diversity of species and the stability of the local environments. The perceived abundance of useful plants is influenced by various factors depending on the use category. For medicinal and timber uses, factors such as the resource’s efficiency, which were not examined in this study, may influence perceived abundance, and warrant further investigation. The proximity of resources to households and the more immediate nature may explain the more accurate perception of the abundance of food and medicinal species, as reflected in the salience index. However, these factors also require further exploration to gain a deeper understanding.

Greater use pressure was observed in the timber category, particularly due to the full use of stems from forest areas in the Queluz community reserve. Nonetheless, these challenges may be mitigated in the short term since the preferred species are perceived as the most abundant in the region, and local programs that encourage the construction of masonry houses support the residents.

In conclusion, this analysis provides valuable insights into the criteria for selecting species in Quilombola communities in Maranhão. Additionally, the analysis must be expanded to include other variables that may influence the perception of local abundance. The complexity of the interactions within socioecological systems is evident, and the criteria analyzed here serve as indicators of conservation practices that are adapted to local needs and realities. These insights can guide sustainable practices within the studied community. Our findings underscore the importance of considering biological diversity, along with the socioeconomic and cultural aspects involved in the use of natural resources, in the development of conservation strategies and the sustainable management of plant resources in local communities.

## Data Availability

This article contains all the data generated and analyzed for its development.

## References

[1] Moerman DE. Symbols and selectivity: a statistical analysis of native American medical ethnobotany. J Ethnopharmacol. 1979;1(2):111–9. 10.1016/0378-8741(79)90002-3. 94415 10.1016/0378-8741(79)90002-3

[2] Gonçalves PHS, Albuquerque UP, Medeiros PM. The most commonly available woody plant species are the most useful for human populations: a meta-analysis. Ecol Appl. 2016;26(7):2238–53. 10.1002/eap.1364. 27755717 10.1002/eap.1364

[3] Albuquerque UP. Etnobiologia: Bases ecológicas e evolutivas. 1st ed. Recife: Editora NUPEEA; 2013. p. 102–26.

[4] Albuquerque UP, Gonçalves PHS, Ferreira Júnior WS, Chaves LS, Oliveira RCDS, Silva TLLD, Santos GCD, Araújo EDL. Humans as niche constructors: revisiting the concept of chronic anthropogenic disturbances in ecology. Perspect Ecol Conserv. 2018;16(1):1–11. 10.1016/j.pecon.2017.08.006.

[5] Silva JB, Silva LB, Albuquerque UP, Castro CC. Bark and latexharvesting short-term impact on native tree species reproduction. Environ Monit Assess. 2018;190(12):1–13. 10.1007/s10661-018-7081-9. 10.1007/s10661-018-7081-9 30470920

[6] Da Silva JPC. (2020) O emprego medicinal de espécies lenhosas protege-as da pressão para usos madeireiros? http://www.tede2.ufrpe.br:8080/tede2/handle/tede2/9324. Acessado em 08 de fevereiro de 2024

[7] Atran S, Medin DI, Ross N. Thinking about biology. Modular constraints on categorization and reasoning in the everyday life of Americans, Maya, and scientists. Mind Soc. 2002;3:31–63. 10.1007/BF02513147.

[8] Atran S, Medin DI, Ross N. Evolution and devolution of knowledge: a tale of two biologies. J R Anthropol Inst. 2004;10(2):395–420. 10.1111/j.1467-9655.2004.00195.x.

[9] Phillips O, Gentry AH. The useful plants of Tambopata, Peru: II. Additional hypothesis testing in quantitative ethnobotany. Econ Bot. 1993;47(1):33–43.

[10] Gandolfo ES, Hanazaki N. Etnobotânica e urbanização: conhecimento e utilização de plantas de restinga pela comunidade nativa do distrito do Campeche (Florianópolis, SC). Acta Botan Brasil. 2011;25:168–77. 10.1590/S0102-33062011000100020.

[11] Cruz MP, Medeiros PM, Sarmiento-Combariza I, Peroni N, Albuquerque UP. “I eat the manofê so it is not forgotten”: local perceptions and consumption of native wild edible plants from seasonal dry forests in Brazil. J Ethnobiol Ethnomed. 2014;10(1):1–11. 10.1186/1746-4269-10-45. 24886156 10.1186/1746-4269-10-45 PMC4038053

[12] Folke C. Resilience: the emergence of a perspective for social–ecological systems analyses. Global Environ Change. 2006;16(3):253–67. 10.1016/j.gloenvcha.2006.04.002.

[13] Faulkner L, Brown K, Quinn T. Analyzing community resilience as an emergent property of dynamic social-ecological systems. Ecol Soc. 2010;23:1–10. 10.5751/ES-09784-230124.

[14] Case RJ, Pauli GF, Soejarto DD. Factors in maintaining indigenous knowledge among ethnic communities of Manus Island. Econ Bot. 2005;59(4):356–65. 10.1663/0013-0001(2005)059[0356:FIMIKA]2.0.CO;2.

[15] Müller-Schwarze NK. Antes and Hoy Día: plant knowledge and categorization as adaptations to life in Panama in the twenty-first century. Econ Bot. 2006;60(4):321–34. 10.1525/maq.2007.21.2.169.

[16] Quinlan MB, Quinlan RJ. Modernization and medicinal plant knowledge in a Caribbean Horticultural Village. Med Antropol Quarter. 2007;21(2):169–92. 10.1525/maq.2007.21.2.169 17601083

[17] Benz BF, Cevallos J, Francisco S, Rosales J, Graf SM. Losing knowledge about plant use in the Sierra de Manantlan biosphere reserve, Mexico. Econ Bot. 2000;54(2):183–91.

[18] Bennett BC, Prance GT. Introduced plants in the indigenous pharmacopoeia of Northern South America. Econ Bot. 2000;54:90–102.

[19] Kala CP. Status and conservation of rare and endangered medicinal plants in the Indian Trans-Himalaya. Biol Conserv. 2004;93:371–9. 10.1016/S0006-3207(99)00128-7.

[20] Albuquerque UP, Soldati GT, Sieber SS, Medeiros PM, Sá JC, Souza LC. Rapid ethnobotanical diagnosis of the Fulni-ô Indigenous lands (NE Brazil): floristic survey and local conservation priorities for medicinal plants. Environ Dev Sustain. 2011;13:277–92. 10.1007/s10668-010-9261-9.

[21] Somnasang P, Moreno-Black G. Knowing, gathering and eating: knowledge and attitudes about wild food in an Isan village in Northeastern Thailand. J Ethnobiol. 2000;20(2):197–216.

[22] Prance GT, Baleé W, Boom BM, Carneiro RL. Quantitative ethnobotany and the case for conservation in Ammonia. Conserv Biol. 1987;1(4):296–310.

[23] Luoga EJ, Witkowski ETF, Balkwill K. Differential utilization and ethnobotany of trees in Kitulanghalo forest reserve and surrounding communal lands, eastern Tanzania. Econ Bot. 2000;54:328–43.

[24] Ramos MA, Medeiros PM, Almeida ALS, Patriota AL, Albuquerque UP. Can quality justify local preferences for firewood in area of caatinga (dryland) vegetation. Biomass Bioenergy. 2008;32:503–9. 10.1016/j.biombioe.2007.11.010.

[25] Albuquerque UP, Medeiros PM, Ferreira Júnior WS, Silva TC, Silva RRV, Gonçalves-Souza T. Social-ecological theory of maximization: basic concepts and two initial models. Biol Theory. 2019;14:73–85. 10.1007/s13752-019-00316-8.

[26] Gaoue OG, Coe MA, Bond M, Hart G, Seyler BC, McMillen H. Theories and major hypotheses in ethnobotany. Econ Bot. 2017;71:269–87. 10.1007/s12231-017-9389-8.

[27] Monteles R, Pinheiro CUB. Plantas medicinais em um quilombo maranhense: uma perspectiva etnobotânica. Revista de Biol e ciências da terra. 2007;7:1–12.

[28] Rodrigues GB (2010) A Conservação da Biodiversidade e da Paisagem em Território Quilombola na Região de Bacabal-MA (Brazil). https://tedebc.ufma.br/jspui/handle/tede/tede/1642 Acessado em: 27 de Julho de 2023

[29] Bernardi CC (2005) Conflitos sócio-ambientais decorrentes da bubalinocultura em territórios pesqueiros artesanais: o caso Olinda Nova do Maranhão. https://bdtd.ucb.br:8443/jspui/handle/123456789/1677. Acesso em 24 de março de 2023

[30] Costa FWD, Pereira PRM. Gestão Socioambiental nas unidades de conservação do Maranhão: Características, conflitos e perspectivas. Geografia em Atos. 2018;1(6):1–24. 10.35416/geoatos.v1i6.5385.

[31] Amorim IFF, Lucena RFP, Almeida EB Jr. Use and conservation of species in an environmental protected area (EPA) in Baixada Maranhense, Eastern AMAZONIA, Brazil: an ethnobotanical study of a quilombola community. Etnobiología. 2023;21(2):86–103.

[32] IBGE (2010) Instituto Brasileiro de Geografia e Estatística. Censo demográfico, v. 2010, 2010. Disponível em: https://censo2010.ibge.gov.br/. Acesso em 3 de dezembro de 2022

[33] Paiva V (2018) Historicidade e identidade quilombola em Anajatuba-Maranhão. http://hdl.handle.net/10314/4580 Acessado em em 28 de maio de 2023

[34] Araújo RIS (2010) Cultura migrante na baixada maranhense. In: História Oral. X Encontro Nacional de História Oral. http://www.encontro2010.historiaoral.org.br/resources/anais/2/1270578017_ARQUIVO_ArtigoABHO.pdf. Acessado em 22 de Junho de 2023

[35] Chagas JDO (2006) Análise da dinâmica de expansão dos sítios urbanos de Anajatuba MA e Pinheiro MA e seus impactos sócio-ambientais: perspectivas de um planejamento sustentável. http://tedebc.ufma.br:8080/jspui/handle/tede/1222 Acessado em 15 e Janeiro de 2022

[36] Carvalho GCA, Ribeiro MHM, Araújo ACAM, Barbosa MM, Oliveira FS, Albuquerque PMC. Flora de Importância Polínica utilizada por *Melipona* (Melikerria) *fasciculata* SMITH, 1854 (HYMENOPTERA: APIDAE: MELIPONINI) em uma área de floresta amazônica na região da Baixada Maranhense, Brazil. Oecologia Australis. 2016;20:58–68.

[37] Somnasang P, Moreno-Black G. Knowing, gathering and eating: knowledge and attitudes about wild food in an Isan village in Northeastern Thailand. J Ethnobiol. 2000;20(2):197–216.

[38] IBGE (2019) Instituto Brasileiro de Geografia e Estatística, Diretoria de Geociências, Coordenação de Meio Ambiente, Áreas Urbanizadas do Brasil. Disponível em: https://biblioteca.ibge.gov.br/index.php/biblioteca-catalogo?view=detalhes&id=2101973 Acessado em 16 de Janeiro de 2023

[39] Albuquerque UP, Ramos MA, Lucena RFP, Alencar NL. Methods for data collection in medical ethnobiology Methods Tech Ethnobiol Ethnoecology. 1st ed. New York: Springer; 2014. p. 15–37.

[40] Kutal D, Kunwar RM, Baral K, Sapkota P, Sharma HP, Rimal B. Factors that influence the plant use knowledge in the middle mountains of Nepal. PLoS One. 2021;16(2): e0246390. 33571303 10.1371/journal.pone.0246390 PMC7877619

[41] Nascimento VT, Pereira HC, Silva AS, Nunes AT, Medeiros PM. Plantas alimentícias espontâneas conhecidas pelos moradores do Vau da Boa Esperança, município de Barreiras, oeste da Bahia, nordeste do Brasil. Revista Ouricuri. 2015;5:086–109.

[42] Termote C, Van Damme P, Dhed’a Djailo B. Eating from the wild: Turumbu, Mbole and Bali traditional knowledge on non-cultivated edible plants, District Tshopo, DRCongo. Genet Resourc Crop Evol. 2011;58:585–618.

[43] Fonseca Filho IC, Bomfim BLS, Farias JC, Vieira FJ, Barros RFM. Uso de recursos madeireiros em duas comunidades rurais de Angical do Piauí/PI, Brazil. Desenvolvimento e Meio Ambiente. 2016;38:593–615. 10.5380/dma.v38i0.44477.

[44] Albuquerque UP, Araújo TS, Ramos MA, Nascimento VT, Lucena RFP, Monteiro JM, Alencar NLA, Araújo EL. How ethnobotany can aid biodiversity conservation: reflections on investigations in the semi-arid region of NE Brazil. Biodiv Conserv. 2009;18:127–50.

[45] Araújo TAS, Alencar NL, Amorim ELC, Albuquerque UP. A new approach to study medicinal plants with tannins and flavonoids contents from the local knowledge. J Ethnopharmacol. 2008;120(1):72–80. 18725282 10.1016/j.jep.2008.07.032

[46] Chettri N, Sharma E. A scientific assessment of traditional knowledge on firewood and fodder values in Sikkim, India. Forest Ecol Manage. 2009;257(10):2073–8.

[47] Wezel A, Lykke AM. Woody vegetation change in Sahelian West Africa: evidence from local knowledge. Environ Dev Sustain. 2006;8:553–67.

[48] Silva JPC, Gonçalves PH, Albuquerque UP, Silva RRV, Medeiros PM. Can medicinal use protect plant species from wood uses? Evidence from Northeastern Brazil. J Environ Manage. 2021;279: 111800. 10.1016/j.jenvman.2020.111800. 33340962 10.1016/j.jenvman.2020.111800

[49] Molares S, Ladio A. Medicinal plants in the cultural landscape of a Mapuche-Tehuelche community in arid Argentine Patagonia: an eco-sensorial approach. J Ethnobiol Ethnomed. 2014;10:1–14. 25159153 10.1186/1746-4269-10-61 PMC4150423

[50] Albuquerque UP, Oliveira RF. Is the use-impact on native Caatinga species in Brazil reduced by the high species richness of medicinal plants? J Ethnopharmacol. 2007;113:156–70. 17616289 10.1016/j.jep.2007.05.025

[51] Ribeiro DA, Oliveira LGS, Macêdo DG, Menezes I, Costa RA, Silva JGM, Lacerda MAP, Souza SR, Almeida MM. Promising medicinal plants for bioprospection in a Cerrado area of Chapada do Araripe, Northeastern Brazil. J Ethopharmacol. 2014;155(3):1522–33. 10.1016/j.jep.2014.07.042. 10.1016/j.jep.2014.07.042 25086410

[52] Corrêa W, Carvalho MWL, Mendes TJ. Atualização da classificação climática e balanço hídrico climatológico no estado do Maranhão. Revista Brasil de Climatol. 2023;32:517–43.

[53] Medeiros PM (2010) Uso de produtos madeireiros para fins domésticos em uma área de Floresta Atlântica no noroeste brasileiro. http://www.tede2.ufrpe.br:8080/tede2/handle/tede2/492. Acessado em 08 de fevereiro de 2024

[54] Leonti M, Nebel S, Riviera D, Heinrich M. Wild Gathered food plants in the European Mediterranean a comparative analysis. Econ Bot. 2006;60(2):130–42.

[55] Rozin P. Acquisition of stable food preferences. Nutr Rev. 1990;48(2):106–13. 2407977 10.1111/j.1753-4887.1990.tb02912.x

[56] Nascimento VT, Lucena RFP, Maciel MIS, Albuquerque UP. Knowledge and use of wild food plants in areas of dry seasonal forests in Brazil. Ecol Food Nutr. 2013;52:1–26. 23802914 10.1080/03670244.2012.707434

[57] Ladio AH, Lozada M. Edible wild plant use in a Mapuche community of northwestern Patagonia. Human Ecol. 2000;28:53–71.

[58] Ladio AH, Rapoport EH. La variación estacional de las plantas silvestres comestibles en baldíos suburbanos de Bariloche, Parque Nacional Nahuel Huapi, Patagonia, Argentina. Vida Silvestre Neotrop. 2002;11:33–41.

[59] Caetano RA, Albuquerque UP, Medeiros PM. What are the drivers of popularity and versatility of medicinal plants in local medical systems? Acta Bot Braz. 2020;34:256–65. 10.1590/0102-33062019abb0233.

[60] Lorenzi H, Matos FJA. Plantas Medicinais no Brazil: nativas e exóticas. 1. rs. Nova Odessa: Instituto Plantarum; 2002.

[61] Gaoue OG, Horvitz CC, Ticktin T. Non-timber forest product harvest in variable environments: modeling the effect of harvesting as a stochastic sequence. Ecol Appl. 2011;21(5):1604–16. 21830705 10.1890/10-0422.1

[62] Gaoue OG, Horvitz CC, Ticktin T, Steiner UK, Tuljapurkar S. Defoliation and bark harvesting affect life-history traits of a tropical tree. J Ecol. 2013;101(6):1563–71.

[63] Ramos MA, Medeiros PM, Almeida ALS, Patriota AL, Albuquerque UP. Can quality justify local preferences for firewood in area of caatinga (dryland) vegetation. Biomass Bioenergy. 2008;32:503–9.

[64] Pedrollo CT, Kinupp VF, Shepard GJR, Heinrichd M. Medicinal plants at Rio Jauaperi, Brazilian Amazon: ethnobotanical survey and environmental conservation. J Ethnopharmacol. 2016;186:111–24. 27058631 10.1016/j.jep.2016.03.055

[65] Gomes TB, Bandeira FPSF. Uso e diversidade de plantas medicinais em uma comunidade quilombola no Raso da Catarina, Bahia. Acta Bot Brazil. 2012;26:796–809.

[66] Reyes García V, Vadez V, Huanta T, Leonard W, Wilkie D. Knowledge and consumption of wild plants: a comparative study in two Tsimane’s villages in the Bolivian Amazon. Ethnobot Res Appl. 2005;3:201–7.

[67] Cruz MP, Peroni N, Albuquerque UP. Knowledge, use and management of native wild edible plants from a seasonal dry forest (NE, Brazil). J Ethnobiol Ethnomed. 2013;9(1):1–10. 24279311 10.1186/1746-4269-9-79 PMC4176140

[68] Aguilar S, Condit R. Use of native tree species by an Hispanic community in Panama. Economic Botany. 2001;55(2):223–35.

[69] Nairne JS, Thompson SR, Pandeirada JN. Adaptive memory: survival processing enhances retention. J Exp Psychol Learn Memory, Cogn. 2007;33(2):263–73. 10.1037/0278-7393.33.2.263 17352610

[70] Shanley P, Luz L. The impacts of forest degradation on medicinal plant use and implications for health care in eastern Amazonia. BioScience. 2003;53(6):573–84.

[71] Addis G, Urga K, Dikasso D. Ethnobotanical study of edible wild plants in some selected districts of Ethiopia. Human Ecology. 2005;33:83–118.

[72] Clement CR, Noda H, Noda SDN, Martins ALU, Silva GC. Recursos frutícolas na várzea e na terra firme em 11 comunidades rurais do Alto Solimões, Amazonas, Brasil. Acta Amazonica. 2001;3(3):521–7.

[73] Costa NG (2019) Etnobotânica de plantas alimentícias utilizadas pelo povo Shanenawa do município de Feijó. http://hdl.handle.net/11449/190910. Acessado em 07 de fevereiro de 2024

[74] Nascimento LAS. Etnografia reflexiva e cartografia da alteridade em comunidades quilombolas: saberes, trajetórias e espaços sociais. Resgate Revista Interdisciplinar de Cultura. 2020;28:e020014–e020014.

[75] Machado CDC, Kinupp VF. Plantas alimentícias na Reserva de Desenvolvimento Sustentável Piagaçu-Purus, Amazônia Central. Rodriguésia. 2020;71:1–12. 10.1590/2175-7860202071076.

[76] Stanley D, Voeks R, Short L. Is non-timberforest product harvest sustainable in the lessdeveloped world? A systematic review of therecent economic and ecological literature. Ethnobiol Conserv. 2012;1:1–39.

[77] Oliveira RLC, Lins Neto EMF, Araujo EL, Albuquerque UP. Conservation priorities and population structure of woody medicinal plants in an area of Caatinga vegetation (Pernambuco State, NE Brazil). Environ Monit Assess. 2007;132:189–206. 17279457 10.1007/s10661-006-9528-7

[78] Bruschi P, Mancini M, Mattioli E, Morganti M, Signorini MA. Traditional uses of plants in a rural community of Mozambique and possible links with Miombo degradation and harvesting sustainability. J Ethnobiol Ethnomed. 2014;10:1–22. 25056487 10.1186/1746-4269-10-59 PMC4112836

